# A new variation of modern prejudice: young Korean men's anti-feminism and male-victim ideology

**DOI:** 10.3389/fpsyg.2023.1230577

**Published:** 2023-10-20

**Authors:** Han Wool Jung

**Affiliations:** Department of Psychiatry, Hallym University Medical Center, Chuncheon-si, Republic of Korea

**Keywords:** motivated social cognition, online group polarization, symbolic racism, insecure attachment, violent video games, pornography

## Abstract

In South Korea, anti-feminism is now rapidly spreading online among young men, who have started to identify themselves as a social minority or “victims” of female power. Despite its ramifications, theoretically, anti-feminism is indistinct from the racism and sexism of White men that emerged more than half a century ago. In view of this, it shares the same root as typical modern racism or sexism, although it appears to be a novel phenomenon. Such a hypothesis was buttressed by quantifying the attitudes of anti-feminists toward various outgroups based on the transference of prejudice theory. Moreover, the subtle sexist undertones hidden in their arguments have been discussed using various psychological theories and empirical data/statistics. Additionally, various potential factors that may shape or accelerate their attitudes or behaviors have been discussed on the basis of the threat-defense theory. Through comprehensive literature review based on this theory, this study proposes the features related to Korean anti-feminism, encompassing behavioral/situational (overindulging violent or degrading Internet contents, verbal aggression), relational/epistemic (ostracism, attachment insecurity, pseudo-rationalism), and group-level (provocative interactions, polarization) attributes, some of which may also influence groups other than young men and ingrain or exacerbate the extreme ideologies of other groups, including young women. Scrutinizing Korean online anti-feminism and male-victim ideology may improve our understanding of the psychological origins of various social extremities or radical ideologies beyond cultural barriers.

## Introduction

In January 2023, the Ministry of Gender Equality of the Republic of Korea announced a plan to revise the current rape law in Korea to incorporate “non-consensual rape,” but this plan was retracted within just a few hours (Lee, 2023a). This plan was an attempt to change the current rape law, which considers only violence or intimidation as requisites for rape or sexual assault, to meet the global standard recommended by the United Nations, but this attempt was soon after overturned by the President's Office of the current conservative government. Moreover, they declared that the law would not be amended under the current government of President Yoon Suk-Yeol, closing off further discussion over this issue (Kim and Lee, 2023). Not surprisingly, such decisions were based on the current government's anti-feminist direction of policy. A floor leader of the ruling party of Korea clarified that there was an anti-feminist basis behind the decision. He asserted that such a bill would trigger gender conflict and increase false rape allegations, claiming that the bill originated from the “distorted consciousness of some politicians to discipline others”; he said that such movements have fostered the gender conflict in South Korea and that they will be careful not to let such a “misadventure” happen again (Ha, 2023).

Most Koreans agree that such a decision from the government was greatly influenced by men in their 20–30s with strong anti-feminist ideology, who are among the primary supporters of the current government (Kim, 2023; Lee, 2023b). Based in online communities, these groups argue that feminist movements have been discriminating against men and forcing people to treat men unfavorably. Their antipathy to feminism is one of the biggest social issues in South Korea today, and there have been several attempts to analyze this phenomenon from diverse perspectives (Kwon, 2019; Kang, 2022a). Their anti-feminism seems especially unusual given that they express their hostility toward feminist movements in strong and aggressive ways, not only stipulating feminism as female egotism but also recognizing themselves as “victims” of female power. Such tendencies were salient in a recent survey conducted by local news outlet SisaIN (Chun and Jeong, 2019). This survey aimed to identify why men in their 20s had exceptionally low approval ratings for the liberal government compared to other groups. The results showed almost 60% of Korean men in their 20s *strongly* agreed that feminism is female supremacy. Many young men were found to think that current society is unfavorable toward men in many ways, including dating and marriage, employment and promotion, and law enforcement, especially for sexual offenses. Based on these results, they argued that approximately a quarter of men in their 20s are “anti-femme warriors” who take anti-feminism and victim ideology as their core identities. They suggested that young Korean men are strongly motivated to think and act based on antagonism toward feminism, believing that they have always been mistreated by a society that is unfavorable to men. Therefore, they have designated this phenomenon as the emergence of the male minority identity. These young men firmly believe that current Korean society discriminates against men, which is far from the so-called “male privilege,” considering themselves innocent victims scapegoated by feminist power.

Their arguments are a frontal attack on the common social perspective that women are a social minority and constitute the strongest backlash ever against women's movements in Korea, which has bewildered the majority of Koreans. In fact, the reactions within Korean society were remarkably poor. Although some feminist scholars have interpreted the male-minority ideology in Korea as a narrative that was triggered by and developed from online communities with misogynistic or hostile sexist views (Kim and Lee, 2017), this consciousness—shared by most of the young men—has led to many Koreans being inclined to hold these views(Cho, 2019). Later, some politicians started to sympathize with their arguments, resulting in the election of a young anti-feminist politician as the leader of the main conservative party of Korea and a candidate who pledged the abolition of the Ministry of Gender Equality as the president of the Republic of Korea (Gunia, 2022; Lee, 2022a). Their claims, which were apparently setting back gender equality in Korea, finally reached mainstream Korean society.

It is a huge paradox that a country with a gender wage gap of more than 30%, which constitutes an overwhelming top rank among the OECD countries (Lee, 2022b), now has a leadership that advocates men's rights rather than women's rights. Feminism has literally become the “f-word” in Korea, and personal attacks and bullying against feminists are becoming a new issue in Korean society (Hines and Song, 2021; Bicker, 2022). Indeed, Korean anti-feminist men assert that the gender pay gap in Korea is derived from women's lack of endeavor or competence and is, therefore, fair (Chun and Jeong, 2019). Furthermore, they say that Korean society retains many issues that are obviously unfair to men, such as military duty imposed only on men (Bicker, 2022; Lee, 2022c). Their claims are nothing more than a collection of personal experiences shared within their own exclusive communities, which are not evidenced by data (Bicker, 2022; Lee, 2022a). Nevertheless, such phenomena cannot by itself prove that the anti-feminism and victim ideology of young Korean men are completely preposterous. They say that the foreign views that aim to “educate” have stemmed from a lack of awareness of the unique circumstances of Korea. They argue that, at least in South Korea, sexism is only an antiquity from the past and is no longer present in current society and that their antagonism toward feminism is a legitimate resistance to a myriad of unjust demands from radical feminists, not sexism or misogyny (Chun and Jeong, 2019; Lee, 2022c).

It is true that Korean society is unique, and there might be unfavorable policies or treatment of men or “misandry,” as they claim. However, at least from a psychological perspective, their claims are completely indistinguishable from the typical patterns of modern prejudice and discrimination, despite their ostensibly unprecedented nature. Although these young men currently have no social power to discriminate against someone, they already have detrimentally sexist views. A recent survey from the Korean Broadcasting System showed that 47% of young Korean men believed rejecting job applications due to the candidate being a feminist to be fair (Song, 2021). Furthermore, 41% of men opposed the legislation of the Comprehensive Anti-Discrimination Act, which corresponds with the Civil Rights Act in the United States. This ideology is comparable to one of White racists and sexists who stood against equality movements in the 1960s–1970s in Western societies. There have been numerous psychological studies regarding this anti-equality backlash, usually among White men. In other words, outside Korea, many scholars and researchers have been observing such groups' modern prejudice and minority ideologies for decades, and they have been studied from the perspectives of racism and sexism. Looking at the astounding similarities between the modern prejudice studied in the West and the anti-feminism and male-victim ideology among young Korean men today, it seems undeniable that sexism is ingrained in their core mindsets.

## History of psychological modern prejudice

As mentioned, the psychological explorations of modern discriminatory ideologies date back to more than half a century ago. Sears and Kinder (1971) discerned some White people's unfavorable attitudes toward Black people even after the advent of the Civil Rights Movement and argued that there are some subtle racist attitudes against Black people that cannot be explained by the existing concept of blatant racism. Through further studies, researchers confirmed that some suburban White people were expressing specific forms of ideological antagonism (*symbolic racism*) against Black people, theorizing its characteristics from the psychological perspective of prejudice (McConahay and Hough Jr, 1976). According to them, some White people's antipathy toward Black rights movements consists of the following arguments: first, racism is not currently as prevalent in the US as in the past; second, today's Black people are enjoying excessive privilege beyond what they deserve; third, Black people's typically lower social status has resulted from their internal problems, not the problems of the social structure; and finally, Black people are continuously demanding special treatment, exploiting equality movements and masking their own incompetence (Sears and Henry, 2003; Tarman and Sears, 2005). Triggered by the studies of symbolic racism in the 1970s, the phenomena of modern racism/prejudice and their origins have drawn the attention of many psychologists and sociologists. Another theory to mention regarding modern prejudice is *laissez-faire* racism, which is a more subtle form of racism based on meritocracy believing that Black people's socioeconomic fails are due to their racial inferiority or lack of effort (Bobo et al., 1997). Such a belief, like symbolic racism, denies the existence of discrimination and shifts the cause of the discriminatory structure to pure personal responsibility. They say that because everyone has equal opportunity and is treated equally in modern American society, it is unfair to blame social structures for their failure (Tarca, 2005).

Therefore, modern racism is considered a union of racial prejudice and ideological beliefs regarding equality of opportunity or fairness of outcome (Bobocel et al., 1998; Sears and Henry, 2003). Later studies regarding modern prejudice have two notable features. First is the expansion of the target of prejudice. Carney and Enos (2017) demonstrated that the survey questions developed for modern racism can also be applied to several outgroups, not only Black people. In other words, the concept of symbolic or *laissez-faire* racism not only applies to racism toward Black people but can also be expanded to prejudice against various minority groups, e.g., ethnic groups other than Black people or homosexuals (Swim et al., 1995; Henry and Sears, 2008). Second is the evolution of the means of expressing prejudice. Berbrier (2000) argued that today's White supremacists and separatists tend to deny their privilege but perceive themselves as “victims,” indicating the emergence of White-victim ideology. Such a claim is deemed a more advanced way of expressing their antipathy or resentment toward equality movements, probably to unfold their claims to themselves or others in a more socially desirable manner. This ideology incorporates the tenets that today's society is the “reverse discrimination” society that treats White people unfavorably, applying “double standards” between White people and other groups or alleged minorities (Boehme and Isom Scott, 2020; Isom et al., 2022). Indeed, such an ideology is also not restricted to racism. These beliefs tend to be shared primarily by White men, being associated with two-fold threats to their masculine ideology as well as to White ideology. According to McIntosh (1988), White men tend to deny their privileges (either to people of color or women), try to rationalize the reasons for the existence of their privileges, and further claim that they are not getting the privileges that they deserve. Similarly, Kimmel (2017) argued that angry White men express their resentment not only toward people of color but also toward other outgroups, such as women, immigrants, and LGBTQ+ groups. Coston and Kimmel (2012) expanded the victim ideology of White men to the “male-victim” ideology, which is identical to the ideology of young Korean men today. They contended that today's White men try to act as victims, veil their actions as Male Rights Movements, and attempt to reverse the apparent power structure and gain social support for their arguments.

## Theoretical examinations of Korean anti-feminism and male-victim ideology

The phenomena introduced so far are recapitulated as the reversal of the sense of privilege and the formation of victim ideology, which is an advanced form of resistance to the equality movement (or the unfairly excessive promotion of minority rights). However, still debatable is whether it is cogent to interpret young Korean men's victim ideology as a sort of prejudice as well, applying the psychological interpretations of modern racism and sexism. In fact, Glick and Fiske's (1996) ambivalent sexism theory, which distinguishes between two ambivalent attitudes within sexism, namely, benevolent and hostile sexism, is not very useful in explaining Korean anti-feminism and male-victim ideology. As revealed in Chun and Jeong's (2019) survey, young Korean men tend to be relatively free from traditional values regarding sex roles or gender stereotypes (i.e., benevolent sexism). The levels of benevolent and hostile sexism among young Korean men tend to be similar to or even lower than older-generation men, despite being higher than those of women of similar ages (Ma et al., 2018; Park and Kim, 2022). Their attitudes toward women are, although extremely hostile toward feminists or women supporting feminism, apparently regarded as far from sexist, especially considering that ambivalent attitudes are considered a hallmark of the subtle forms of contemporary sexism (Glick and Fiske, 2011; Connor et al., 2017).

However, it is notable that today's antagonism toward feminists involves a typical repertoire of labeling deviant behaviors (Schur, 1971; Link and Phelan, 2013). Such labeling does not only target radical feminists or social extremists, it also eventually puts pressure on women in general to endorse feminist arguments. In fact, these phenomena have long been observed and scrutinized in various fields, including communism (“McCarthyism”; Johnson, 2006), LGBTQ (Callis, 2013), mental disorders (Link and Phelan, 2013), and women (Schur, 1984). Although they overtly seem to blame only feminists or “female supremacists,” they aim to influence women of similar ages to them, who may either be their friends or rivals. Indeed, their ambivalence appears here. They outline the “model women” that correspond to their ideal images of women (even if they might be somewhat different from conventional images of virtuous women) and try to control the same-aged women by praising ideal women and derogating women who deviate from these standards (Papanek, 1994). They attempt to support the social archetype of “good” and “bad” women and reinforce masculine social systems by controlling women with this dichotomy; such elements are considered one of the prominent principles of sexism (Bareket et al., 2018). They may also adopt somewhat strategic maneuvers to dominate women using this dichotomy: praising women who keep their distance from feminism as “sensible,” they implicitly (or sometimes even explicitly) demarcate between model and deviant women. Such a phenomenon has also been observed in other targets of prejudice, for instance, exalting Asians who are stereotyped as being obedient to the system may proliferate racist threats toward Asians and other ethnic groups (Kramer, 2003; Chou and Feagin, 2015). Similarly, highlighting exemplary immigrants may entail a masked intent to implicitly underline the illegitimacy of some immigrants or refugees (see Petterson, 1966; Chu, 1997).

These forms of labeling “some” women or strategic sexism are also observed in contemporary Korean far-right online communities. Um (2016) analyzed the posts on a Korean extremist online community and concluded that although they explicitly argue for equal distribution of responsibility between men and women, misogynistic men practically dehumanize/objectify women, value traditional gender roles of dominant men and obedient women, and, finally, exhibit their misogyny by glorifying subservient and docile women who fit such gender stereotypes. However, one limitation is that this theory cannot explain why anti-feminism and male-victim ideology became the mainstream beliefs among young Korean men. Despite the evidence that extreme anti-feminists have traumatic experiences or obsessions regarding social interactions with women (Chun and Jeong, 2019), Choi's (2018) narrative study revealed that although young Korean men have some expectations of traditional gender roles, they also perceive such roles as a burden. Whatever its motives were, they seem to have repulsion as well as approval for traditional gender roles. In this respect, perhaps the ambivalence of young Korean men is towards their internal minds rather than women. Choo's (2021) qualitative study implies that the conflict of identity derived from pressures of gender roles and competition appears in several different categories, not merely unilateral antagonism toward women or feminism. Although some extremists may substitute the frustration experienced by gender role pressure or failure in competitions or romantic relationships with women with overt or ambivalent misogyny, most young men do not seem to express it beyond internal conflict.

## Evidence for transference of prejudice

Despite the paucity of evidence suggesting that young Korean men's prevailing anti-feminism and male-victim ideology are the ambivalent attitudes being emphasized in many sexism theories, evidencing that most young Korean men's attitudes are related to prejudice generalizable to various targets, as other modern racism/sexism is less challenging. If their antipathy toward feminism is only restricted to “excessively radical and female-supremacist ideologies” as they claim, their hostile attitudes will only be shown to feminists but not to other outgroups. In contrast, according to the recent transference theory of prejudice, certain types of prejudice can transfer into other types of prejudice as well, due to similar psychological motivations for prejudices against different groups (Sanchez et al., 2017). That is, prejudice or its psychological underpinnings do not limit prejudice only to specific groups but lead people to regular negative perceptions or derogations toward multiple outgroups. Therefore, if their attitudes are not specific to feminists but general prejudice, they should have been transferred to other outgroups or minorities as well. In fact, phenomenological evidence regarding it is relatively obvious. For example, Lee Jun-Seok, who was elected as the leader of the now-ruling People Power Party in 2021, attracted attention again the following year by making vigorous statements against the subway protests of people with disabilities, promoting negative opinions about the protests among many young men (Lee and Kim, 2022). Indeed, today's Korean society is rampant with hostility between groups, enough to be called the “age of hatred,” and many people agree that such hate speeches are dominant among young generations who are currently the most active in online communities and leading Internet cultures (Kim et al., 2020; The National Human Rights Commission, 2021).

**Figure 1:**
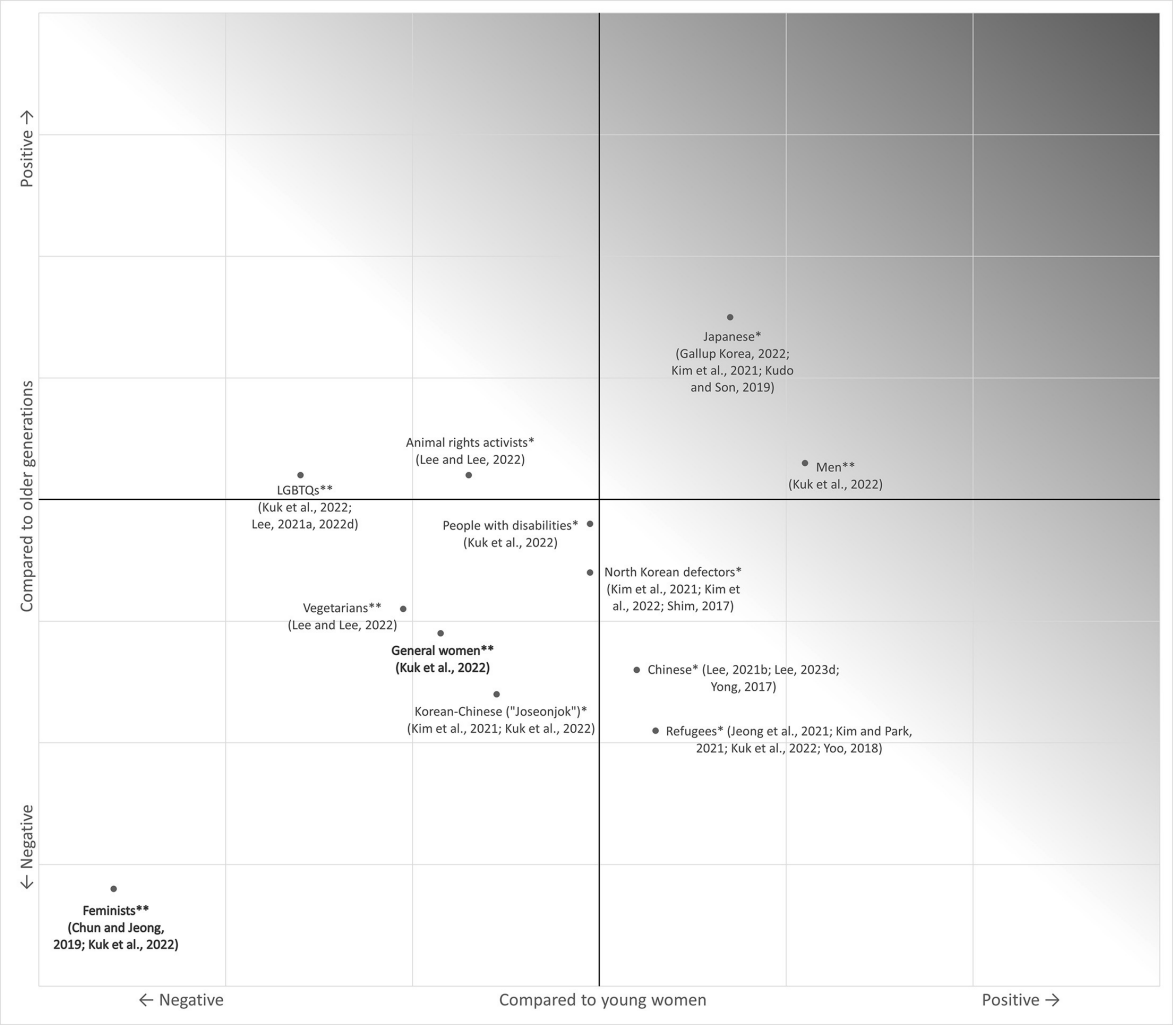
Young Korean men's relative likability toward outgroups. **High-level empirical evidence, *low-level empirical evidence. This is just a rough estimation based on various types of information. Evidence of high-level credibility refers to mass empirical statistics or two or more consistent statistics for both dimensions. Low-level credibility refers to the evidence that it is difficult to reliably estimate both dimensions, based on only one unreliable/low-quality statistic, that the effects can only be estimated indirectly, or with multiple but inconsistent statistics. However, the higher level of evidence may not indicate that the locations on the coordinates are more accurate.

Figure 1 visualizes the attitudes toward outgroups of young Korean men estimated through various sources, compared to older generations and young women. In general, young Korean men generally show more negative attitudes toward outgroups than women of the same age or older generations, despite the particularly conspicuous attitudes toward feminists. They are hostile toward various minorities/outgroups, especially compared to older generations, except for Japanese people, whose likability is associated with political conservatism (Lee, 2023c). Despite the most explicit and salient attitudes toward feminists or “women's power,” they have prevalent hostility toward sundry outgroups. In particular, such attitudes cannot be fully explained only by the “consciousness of fairness” that they emphasized; observation of derogations toward the groups unrelated to salient competition/conflict or the groups based on innate characteristics suggests that their behavior should be interpreted from the perspective of more global and typical prejudice.

However, there is some room for objection here. Although young men's attitudes toward outgroups seem clearly negative compared to older generations even after removing outliers such as feminists and Japanese people, their attitudes might not seem significantly more negative than those of young women when removing the groups related to gender issues (e.g., feminists, other gender groups, the LGBTQ community). In this case, we may hypothesize that the overall negative attitudes toward outgroups came from the generation effect rather than gender differences, making the current issue of outgroup exclusiveness the problem of the “MZ Generation,” including young women as well as young men (Cho, 2022). Expanding this viewpoint, there is strong outgroup prejudice pertaining to both sexes within the younger generations, and it would be sound to interpret this phenomenon as “gender conflict” rather than mere sexism or misogyny. This argument is ostensibly reasonable, but there are some points to clarify. First, regarding gender issues, young women are not as extreme as young men. In Chun and Jeong's (2019) survey introduced above, the proportion of young women classified as radical feminists was <1%, whereas the proportion of young men classified as extremist anti-feminists or “anti-femme warriors” was a whopping 26%. Kuk et al. (2022) reported that most young women tended to reject radical arguments related to gender issues more than older generations and even to a similar extent to young men. In contrast, one study analyzed comments from major online communities in Korea with an artificial-intelligence-based big data processing algorithm and concluded that misogynistic remarks had been increasing before the emergence of feminism as a major social issue, whose slopes were also not significantly different from recent anti-feminist remarks (Park, 2022). This also counterevidences the claim of most Korean anti-feminist men today that the emergence of radical feminism triggered their anti-feminist backlash: at least for gender issues, the alleged gender conflict should be pondered from the perspective of structural misogyny or sexism, rather than the vehement combat between the two equally extreme groups.

Second, South Korea is predominantly exclusive to outgroups. Multiple statistics suggest that prejudice against several minority groups is far stronger in Korea compared to most other countries (Haerpfer et al., 2020; U.S. News, 2022). Considering that most Koreans are generally prejudiced against outgroups, even stronger negative attitudes than other Koreans will indicate their extreme levels of exclusiveness, which is especially ominous compared to foreign countries or global standards. As discussed, this phenomenon also affects young women. To illustrate, there was a strong movement against Muslim refugees recently, which has become a major social issue regarding multiculturalism in South Korea, and one of the groups leading this movement consisted of radical feminist forces mainly composed of young women (Kim, 2018; Lee, 2020). The hostility of young women toward outgroups is as strong as that of young men if irrelevant to gender issues, suggesting that young women also have extreme levels of prejudice toward some outgroups. To summarize, prejudice or sexism against women as a gender issue does exist, prevailing especially among young men, indicating that there are some precursors of prejudice against various outgroups that influence not only young men but also young women. Such precursors may have been stronger for young men, which made them extremely exclusive regarding the most salient gender issues, but some would also have affected young women and made them exclusive toward other outgroups.

## Potential psychological underpinnings of Korean anti-feminism and male-victim ideology

young Korean men's anti-feminism and male-victim ideology today are indistinguishable from the modern racism and sexism in Western societies that have been theorized for more than a half-century. Despite some uncertainties, the case in Korea also seems to have been derived from attitudes related to prejudice, which are identical to Western modern racism/sexism. As a social psychologist, my next goal will be to find the psychological origin of this case. Unfortunately, however, studies on the prejudice of today's young Korean men mostly remain at the level of phenomenological analyses, with few studies focusing on the psychological frameworks. Nevertheless, it is possible to contemplate the psychological backgrounds of many young Korean men's prejudices today depending on multimodal theories or evidence. Hence, here, we delve deeper into the etiology of young Korean men's anti-feminist and minority ideology through various psychological theories and discuss their plausibility based on empirical evidence.

To provide social psychological explanations for extremist ideologies, the easiest theory to present would be that of defensive reactions to threats. Humans are inherently designed to automatically produce defensive reactions when they perceive external (or sometimes internal) uncertainties that contradict their goals. In other words, salient circumstantial threats and the perceptions that such threats challenge their needs induce physical/emotional anxiety or cognitive dissonance, which motivates people to relieve it by changing their attitudes or behaviors (Nash et al., 2011; Reiss et al., 2021). Studies in this area have been conducted in diverse forms in psychological fields with varied nomenclature, but Jonas et al. (2014) integrated such theories with biological/neuroscientific explanations and managed to establish a general process model of threat and defense. According to them, perceptions of threat first create *proximal* defensive reactions, which are relatively immediate physical or emotional anxiety reactions composed of vigilance, arousal, avoidance, or the interactions between them. The dissonances created by proximal defense stimulate people to produce more *distal* defensive reactions: Such reactions signify more active or approach-oriented responses to dissolve the dissonances. The distal defense encompasses either individual (personal) or interpersonal/group (social) levels and either pursues tangible incentives (concrete) or intangible changes in attitudes or ideologies (abstract). Finally, the entire process of proximal and distal defense reactions is moderated by the individuals' underlying dispositional motivations.

**Figure 2:**
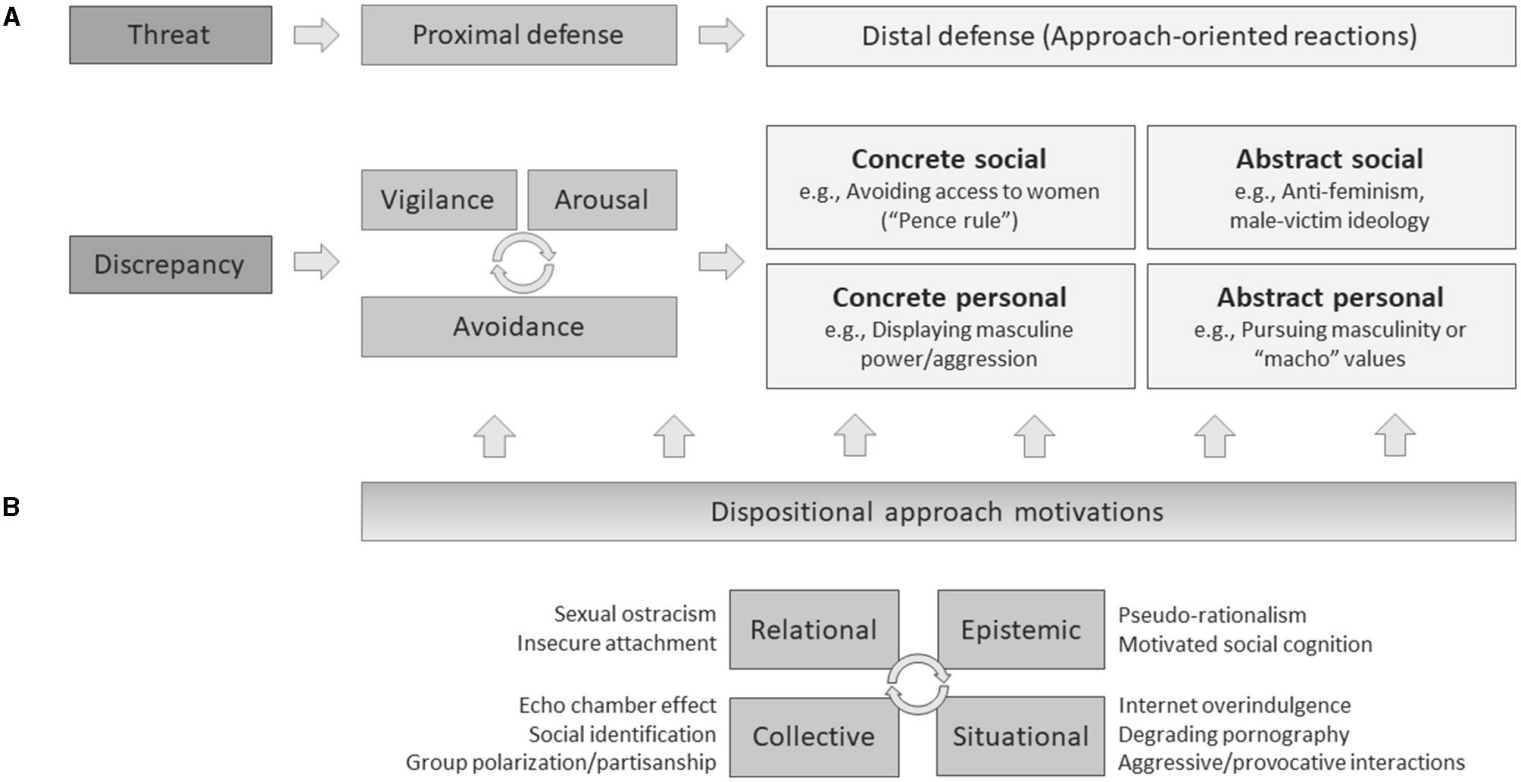
Illustration of psychological model for anti-feminism and male-victim ideology. Adapted from Jonas et al.'s (2014) illustration of the anxiety-to-approach model of threat and defense. Examples described in **(A)** are only anecdotal explanations based on the hypothesis of feminism as a threat to masculinity. **(B)** describes possible motivational, social-cognitive, or behavioral bases that may trigger or accelerate anti-feminism and male-victim ideology as defensive reactions, and they do not typically presume feminism as a masculinity threat. Adapted from Jonas et al. (2014) with permission from Elsevier.

According to this theory, Korean anti-feminism and male-victim ideology are only one form of reaction to a certain threat (i.e., abstract-social). The presence of threat (and the perception of such threat) can engender various types of defensive reactions, which applies to reactions to the threat of feminism as well. In fact, the theory that explains anti-feminist backlash as a reaction to the threat of feminism to masculinity has widely been accepted by feminist scholars as well as psychologists (Faludi, 2006). Figure 2A describes examples of defensive reactions to the feminism threat, positing this theory. However, even positing this theory, at least three points should be noted. First, not all people or men regard feminism as a threat to their masculinity. This applies to anti-feminist men as well as pro-feminist men; as discussed above, young Korean men tend not to strongly pursue conventional masculine roles or values, at least explicitly, which may negate the theory of threat to masculinity. The feminist threat may not make all people defensive (at least in terms of masculinity), and there should be some other origins beyond the threat to masculinity. Second, thinking conversely, threats irrelevant to feminism or gender issues may also be the origin of anti-feminism. In practice, ingroup favoritism and exclusiveness toward outgroups are typical reactions to general threats, such as mortality salience (Li et al., 2015). Moreover, considering the social-cognitive mechanism of attitude change, the source of the threat and the target of its reactions may not always be parallel (see Gawronski and Bodenhausen, 2006). In other words, anything known as difficulties of today's young generations in Korea, e.g., frustration, relative deprivation, or the sense of alienation caused by the hostile social structures (Yeom and Nam, 2021), could be a threat that becomes the origin of outgroup prejudice or anti-feminism. This seems somehow similar to the “unfairness” discourse mentioned above. However, in general, the threat-defense theory does not take into account whether the threat perceptions and the sense of deprivation or alienation here are genuine or distorted consciousness. In other words, regardless of whether society really is hostile toward them or whether it is merely a false consciousness, the perception that the threat is hostile (and therefore, that it contradicts their goals) can by itself trigger threat processing; therefore, this is wholly a matter of internal coping.

Finally, and most importantly, even perceiving feminism (or something spuriously admissible as feminist things) as a threat does not seem to always result in anti-feminist defensive reactions. In fact, individuals have various unique modalities in coping with threats, encompassing every domain of human behavior as well as social/collective ideologies. Furthermore, even assuming that such reactions are predictable, at least in the realm of social attitudes, they seem to move in a way to reinforce existing beliefs rather than to lead people in certain directions. Although some studies conclude that threat induces people to act in a way to maintain the *status quo* (Jost et al., 2003; Nail et al., 2009), multiple studies suggest that priming mortality salience can make liberals more liberal as well as make conservatives more conservative (Greenberg et al., 1995; Weise et al., 2008). Considering that gender issues are germane to political orientations in Korea as well, such a mechanism of threat and defense may also explain the way in which feminism becomes more radical; this theory is basically for the general behavior of laypeople, which may not be very useful to account for certain groups or inclinations of behaviors only. Although it is true that the overall extremity of today's Korean society is also influential, analyzing these phenomena only by this framework may ignore a myriad of core properties of these extremely conspicuous attitudes. Specifically, although blatant misogyny had increased among Korean online communities even before the emergence of feminism as a major issue, this approach can only explain the reactions after feminism became salient. Indeed, there should be complex mechanisms incomprehensible with a simple diagram from the feminist threat to defensive reactions. Moreover, some or most of the antecedents should not directly relate to feminism/feminist threat or were developed before the emergence of feminism, although they may have facilitated the severity of the backlash among young Korean men after the emergence.

Consequently, despite the robust base of this model, only naively applying the threat-defense model to Korean anti-feminism and male-victim ideology lacks not only theoretical grounds but also empirical evidence. Therefore, here, we focus on the factors that uniquely explain their behavior while maintaining the basic theoretical framework of threat and defense. Such factors stand for diverse underpinnings concerned with every domain of defensive reactions to threat, suggesting the attributes that Jonas et al. (2014) named “dispositional approach motivations.” However, the attributes covered here are not limited to dispositional factors but encompass various factors in all areas of life, including social relations, epistemic motives, circumstantial influences, or collective identities (see Figure 2B). Nevertheless, this approach also postulates the predisposed uniqueness of Korean anti-feminists, which are involved in their regular social cognition, appraisal, and attitude formation (Kruglanski, 1990), and I hypothesize that such systematic inclinations will uniquely explain the behavioral patterns of Korean anti-feminist young men as well.

## Insecure attachment

Attachment is one of the factors that Jonas et al. (2014) emphasized as an underlying moderator of defensive reactions to threat. According to the attachment theory, the parental bond experienced in childhood affects almost every relationship after people grow up: it changes personal development, creates fundamental schemas for social relationships, and exerts the most decisive influences on the entire stages of one's life (Bowlby, 2008). It applies to defensive reactions as well. Nash et al. (2014) argued that those who are securely attached have temperaments to prevent excessive anxiety activation in their neural networks even in threatening situations, which prevents immoderate or maladaptive defensive reactions. In contrast, those with insecure attachment cannot control their anxiety well and, therefore, are vulnerable to dysfunctional behaviors in intimate relationships, including dating or romantic relationships. In fact, attachment insecurity is associated with the tendency of casual sex or negative affect in sexual relationships (Gentzler and Kerns, 2004). Insecure attachment also predicts violence or abuse in partner relationships (Dutton et al., 1994; Oka et al., 2014).

However, more important is that attachment affects group-level interactions as well, not only interpersonal relationships. Multiple studies suggest that attachment can explain social extremity or prejudice as well as individual malfunctioning (Carnelley and Boag, 2019). Mikulincer (1997) reported that adults' insecure attachment is associated with cognitive closure or stereotypes in judgment and decision-making processes. Experimental research also supports this: Saleem et al. (2015) demonstrated that priming secure attachment can reduce negative views toward outgroups. Weise et al. (2008) proposed that secure attachment prevents political polarization when threat is salient, whereas Boag and Carnelley (2016) suggested that insecure attachment leads to prejudice by decreasing empathy. Moreover, attachment style seems especially relevant to sexism: the influence of attachment on romantic relationships may also be valid to group-level interactions or attitudes regarding sexual themes. Fisher and Hammond's (2019) meta-analysis presented that attachment anxiety and avoidance are related to both benevolent and hostile sexism, and such relationships were stronger among men compared to women. Hart et al. (2012) also reported that attachment insecurity may affect benevolent and hostile sexism, mediated by romanticism. However, a caveat in this study is that the mediating effect of romanticism was usually concerned with benevolent sexism, whereas hostile sexism was more affected by factors related to prejudice such as social dominance orientation. In other words, the relationship between attachment insecurity and antagonistic sexism might be more associated with social values than romantic relationships; the group-level effects of insecure attachment might be in different domains from the individual- or relational-level effects.

## Sexual ostracism

However, although the connections between attachment and sexism are not well-explained in romantic relationships, it does not, by itself, negate the association between romantic experiences and sexism. Arendt (1973) argued that loneliness and social isolation may be the origin of authoritarianism and totalitarianism. Indeed, the need for belongingness is one of the most fundamental human needs, and the condition of loneliness is considered a serious threat to survival, motivating humans to promptly address it (Cacioppo et al., 2014). Multiple studies suggest that loneliness is likely to result in aggression toward others or outgroups (Buelga et al., 2008; Odaci and Çelik, 2013) and may cause neutral behaviors to be interpreted as negative or hostile, which can increase not only individual anxiety but also social hostility (Chen et al., 2020; Trotta et al., 2021). There is some evidence that the anti-feminism of young Korean men might be associated with loneliness or sexual ostracism. Choo (2021) classified two types of Korean men with highly anti-feminist attitudes, one of which had generally fewer friends than other types, and the other used to socialize mostly with boys and had fewer female friends than other types. Although these differences were not decisive, it was one of the strongest predictors of the differences between the classified types. However, in general, evidence suggesting the causal relationship between loneliness and prejudice is lacking. Floyd (2017) studied the relationships between loneliness and xenophobia/right-wing authoritarianism and found significant but trivial correlations. Nevertheless, considering Cacioppo et al.'s (2014) argument that loneliness is a physiological and evolutional mechanism for human survival and reproduction, it is a possible conjecture that repetitive experiences of rejection in dating and concomitant senses of frustration/alienation may lead to hostile and aggressive behaviors toward the opposite sex. In reality, the extreme online ideology or culture of men alienated from dating (“involuntary celibates” or incels) is a social issue even outside of Korea, and academic research regarding them is also increasing in diverse fields (O'Malley et al., 2022; Sparks et al., 2022). Chun and Jeong (2019) also observed some distorted perceptions regarding romantic relationships among young men with extreme anti-feminist and male-victim ideology.

Nevertheless, this does not necessarily indicate that Korean anti-feminism and male-victim ideology have been steered by “naturally selected” young men. Outside of Korea, there is a study that incel online activity is higher in regions where the proportions of male populations are higher than females (Brooks et al., 2022). However, there is insufficient evidence that the lack of physical experience with women increases men's anti-women bias. For example, de Lemus et al. (2010) demonstrated that the level of adolescents' romantic relationship experiences was positively associated with sexist beliefs, indicating that their desire to seek romantic partners increased hostile attitudes toward the opposite sex. Fisher and Hammond's (2019) meta-analysis also showed that (i) the level of hostile sexism was not higher for men who were not in romantic relationships compared to men in committed relationships and (ii) the level of benevolent sexism was rather higher for men in romantic relationships. Although this is only a descriptive relationship without demographic control (e.g., age), they also argued that the association between avoidant attachment and hostile sexism only appeared among men in romantic relationships. Even if hostile attitudes toward women did indeed stem from sexual ostracism, they do not seem to stem from the absence of dating experiences *per se*. However, the accumulated experiences of failure and frustration in relationships with the opposite sex may have strengthened their sexist perceptions, and further studies are needed to identify this, especially among Koreans.

## The Internet

Today, the Internet is a medium of various extreme and discriminatory ideologies, not limited to anti-feminism and male-victim ideology in Korea. Violent claims and hate speech in online spaces and social media, especially among adolescents and young adults, are observed not only in Korea but worldwide (Hawdon et al., 2015; Costello et al., 2020). Phenomenologically, it seems obvious that the Internet has become a channel for extremism (Gaudette et al., 2020), but there are various hypotheses as to its reasons. One of which is linked to the loneliness theory above: simply, those who feel ostracized or depressed are vulnerable to problematic Internet use (Caplan, 2003). Indeed, one study reported that problematic Internet use is associated with low empathy (Melchers et al., 2015). However, in general, Internet usage *per se* does not seem to be inducing social dysfunctions (Shklovski et al., 2006). The prevalence of extremism on the Internet is likely to be the result of complex interactions between the uniqueness of online circumstances and the individual/collective characteristics vulnerable to extremism, especially when reacting to various social threats, rather than the mere environmental effects of the Internet itself (Vergani et al., 2020). Therefore, the relationship between the Internet and extremism should be comprehensively explored from many different perspectives, covering almost all areas of social psychology, including social learning and communications, maladaptive behaviors, and group interactions. The current study covered a comprehensive review of these factors. Consistent with the “prejudice of young generations” theory mentioned above, most of the factors presented here seem more vulnerable to young generations, and some are even more vulnerable to young men. This may explain why most young men in Korea have strong sexist prejudices and young women also have non-gender-related prejudices.

### Overindulgence

Researchers have argued that excessive or pathological usage of the Internet or online video games may increase maladaptive behaviors. They argue that many young people today are overly using or are “addicted” to the Internet or online games, and violent online content may increase their aggressive behaviors. Still, there is no consensus in academia as to whether pathological Internet use or gaming adversely affects mental health. However, to conclude, the argument that pathological Internet use or gaming is innocuous is a minority opinion in academia. Ferguson and Colwell (2020) reported that 60.8% of scholars believe pathological gaming can lead to mental health problems, whereas only 30.4% were skeptical about that. Since Anderson et al. (2010) released a famous meta-analytic review suggesting that violent video games increase aggression, some researchers have criticized this research by arguing that the effect sizes were overstated (e.g., Ferguson, 2015; Hilgard et al., 2017), but this association has generally been reproduced until recently, including in longitudinal studies (Mathur and VanderWeele, 2019; Burkhardt and Lenhard, 2021). In contrast, the skeptics have mainly focused on peripheral criticisms such as methodological issues (Carnagey and Anderson, 2004), individual bias of the researchers of pathological gaming (Ferguson and Colwell, 2017), emphasis on social/environmental contexts of pathological gamers (Jeong et al., 2019), and the arguments that its severity is “exaggerated” (Ferguson et al., 2011; Ferguson, 2015). Griffiths et al. (2017) argued that many criticisms of pathological gaming do not properly distinguish the difference between gaming as healthy leisure and problematic gaming activities.

Indeed, skeptics also cannot deny that the phenomenon of pathological Internet use or gaming is real, even if the negative effects of the Internet or gaming itself are not particularly significant. Griffiths et al.'s (2012) review concluded that the prevalence of medically diagnosable video game addiction ranges from 1 to 20–30% by country. In particular, in this review, the rates of prevalence were generally higher for men compared to women, indicating that young men are more vulnerable to the adverse effects of gaming, including aggression or hostile behaviors. In addition to phenomenological and empirical evidence, theoretical or neuroscientific evidence has also been sufficiently accumulated to interpret the negative impacts of the Internet/gaming or their overuse. This theory and evidence encompass traditional vicarious learning theory (Bandura, 1978; Allan, 2017), aggression models (Bushman and Anderson, 2002; Werner et al., 2010), and neurological models regarding the relationship between behavioral addiction and aggressive behaviors (Hahn and Kim, 2014). In contrast, the hypothesis or popular awareness that violent video games allow people to relieve or vent their stress or anger and reduce future aggressive behaviors in the real world (“catharsis theory”) is largely untrue based on recent studies (Bushman, 2002; Schaefer and Mattei, 2005).

However, the studies mentioned so far may not confirm the negative effects of pathological Internet use or gaming. Briefly speaking, they can only evidence that violent video games may increase people's aggression, probably to a small extent, and the pathological use of the Internet/video games may influence some people's mental health, which is still controversial. Moreover, positing the negative effects of the Internet can neither justify the registration of gaming disorder to the 11th revision of the International Classification of Diseases (ICD-11) of the World Health Organization nor show that pathological gaming can lead to anti-feminism or male-victim ideology among young men in South Korea. Further evidence is needed to clarify the negative impact of Internet overuse or pathological gaming, either individually or socially/collectively.

### Pornography consumption

Unlike the controversial issue of Internet overindulgence, there is a seemingly more destructive and sexually relevant problem on the Internet: porn consumption. Today, more than half of men and 20%−40% of women are estimated to be consuming pornography on the Internet (Zattoni et al., 2021). Pornography may have a bigger impact than other content on the Internet or video games, especially related to gender issues: it ruins relationships with romantic partners (Bridges et al., 2003), is associated with male impulsivity (Antons and Brand, 2018), and when overused, causes frontal lobe dysfunctions, which may lead to pathological behaviors such as violence against women (Hilton and Watts, 2011). young Korean men are no exception to the problem of Internet pornography consumption. According to recent statistics, the exposure to Internet pornography in Korea was highest among young men, who also had the highest tendency to believe that watching pornography is not a moral problem (Park, 2019). Seo (2020) argued that Korean adolescents are pervasively exposed to pornography on the Internet, whether voluntarily or not, putting them at risk of developing problematic behaviors related to sexual violence.

In general, however, there is limited evidence of the negative impacts of pornography, and evidence of the effects of pornography on sexist prejudice is even more limited. Criticisms of the studies on the individual/social effects of pornography consist of their methodological issues, ambiguity in discriminating it from sex addiction, and the effects or outcomes considered non-pathological (Ley et al., 2014; Duffy et al., 2016). For these reasons, some researchers argue that pornography is in fact benign (Ley et al., 2014). Seto et al. (2001) argued that the effect of pornography on sexual aggression is significant only for already predisposed men, suggesting that pornography consumption may only be a factor that moderates sexual aggression or even a mere phenomenological outcome, rather than the cause of sexual aggression or violence. In other words, to link porn consumption with young Korean men's anti-feminism, it seems necessary to focus on the specific forms or cultures in which they consume pornography rather than the effect of pornography *per se*. Indeed, as one of the typical male-predominant cultures, pornography often contains content that is sexually aggressive toward women or exaggerated masculine identities (Fritz et al., 2020; de Heer et al., 2021). That is, at least some pornography is, by itself, an expression of sexist views or prejudice, degrading women as sexual objects. The sociocultural contexts regarding the sexual objectification of women in pornography may relate to degrading attitudes toward real women among pornography users (Attwood, 2004; Willis et al., 2022). Skorska et al. (2018) found that pornography can increase men's sexual objectification of women, sexist beliefs, and discriminatory attitudes toward women; these links were mostly found with sexually degrading pornography (i.e., pornography characterized by dehumanization/debasing of women) rather than in general pornography. Consistent results have been observed among Koreans as well. Willis et al. (2022) reported a strong association between pornography use and sexual objectification among Koreans. Also, interestingly, the interaction effect of gender and pornography use on sexual objectification was observed only among Koreans and not among other nationalities, suggesting that Korean men can be especially vulnerable to sexual objectification when watching pornography.

### Provocative culture

The Internet also has its uniqueness in terms of interactions and social activities, as well as the individual-level influences discussed above. The uniqueness of Internet cultures can either be the fundamental structure of online spaces (e.g., the fragmentary nature of personal relationships) or the communicative preferences that have been made by previous and current Internet users. Firth et al. (2019) discussed that the Internet causes an increase in people's attention and memory burden due to the flood of information they are exposed to, pressuring people into automatic and immediate judgment and decision-making. Furthermore, they argued that the online networks' immediate feedback on successes and failures in social relationships affects the users' self-esteem, causing people to devote their online activities mostly to managing social impressions. To clarify, the Internet makes people “unthink” and forces judgment based on stereotypes and prejudice, and the existence of “likes” and social comparisons impairs diversity in social relationships but makes online relationships like a unidimensional and vertical hierarchy. In particular, Firth et al. (2019) argued that the social-cognitive adverse effects of the Internet are greater in young people: experiences of immediate feedback on the self-esteem of adolescents not only lead to mental health problems such as a sense of isolation, depression, and anxiety but also aberrant behaviors such as cyberbullying. Another issue is that the Internet overrepresents some extreme opinions or ideologies. Yun et al. (2019) analyzed the editing history of Wikimedia and concluded that the information or opinions produced on the Internet are made only by a small proportion of people, arguing that the oligarchy of information makes the online environments misrepresent extreme ideologies rather than welcome diverse viewpoints. Due to these phenomena, the Internet is flooded with people willing to beat others, provocateurs, and those who want to stand their ground, pushing away tolerance, empathy, diversity, or deliberation. Furthermore, the structure of the Internet rewards such people rather than punishing them, creating online environments full of aggressive and extreme claims (Koehler, 2014; Bryant, 2020).

#### Anonymity and deindividuation

Anonymity is one of the essential parts of explaining the uniqueness of online communications. Because anonymity and its accompanying deindividuation decentralize individual responsibilities and free people from the social consequences of their behavior, individuals under anonymity tend to easily become less self-controlled, sometimes leading to radicalized, impulsive, or aberrant behaviors (Zimbardo, 1969). This can also explain why hate speeches are prevalent in online spaces. As anonymity and deindividuation grant exemptions from the responsibility for violent languages, online spaces are often used as the pathway for expressing discomfort/furtive desire and aggression (Lowry et al., 2016; Zimmerman and Ybarra, 2016). The effect of anonymity and deindividuation on bad comments on the Internet has recently been illustrated in Korea. Since Korea's largest web portal, Naver, has recently decided to open all commenters' comment history to the public, the average number of comments per day has decreased by 38%, and notably, malicious comments have dropped by 70% (Jeong, 2020). However, the caveat is that anonymity alone may not always lead to extremity. Rösner and Krämer (2016) suggested that the negative effects of anonymity only apply when the desire for expression under anonymity is justified in the collective atmospheres that allow for each other's aberrant behaviors. Similar to Internet overuse and pornography consumption, as described above, anonymity should also be seen as a moderator or risk factor rather than a direct cause of extremism. However, after group homogenization or polarization has progressed to a certain extent, anonymity can become a channel through which people no longer hesitate to express extreme or destructive claims, which will be explained below.

#### Verbal aggression

The prevailing aggressive culture on the Internet may be either the product of dispositional attributes of Internet users or the circumstantial effects derived from the violent content on the Internet, as discussed above. In any event, extreme or prejudiced arguments on the Internet are, by themselves, germane to verbal aggression. Although not all types of aggression are related to prejudice, online hate speech or offensive language is the real issue, being an urgent and incessant social problem in a wide range of fields (Ştefăniă and Buf, 2021). However, although trait aggression or aggressive culture on the Internet is supposed to be a risk factor for online sexist speech or misogyny, evidencing these relationships has clear limitations with the current knowledge. First of all, quantifying the “aggressive online culture” is naturally bound to be subjective, mostly due to the vagueness in the determination of extreme or hate speech (Pohjonen and Udupa, 2017). In other words, it is hard to objectively identify whether the anti-feminist male communities in Korea are more aggressive than other communities. Nevertheless, it is possible to investigate whether men, especially young men, generally have more verbal aggression than women. Because aggression is linked to the activity of androgens, boys' aggression is generally more prominent than girls', especially during their childhood development (Ramirez, 2003). However, it is unclear whether this can be extended to verbal aggression on the Internet. Considering that men's aggression is mostly expressed in a physical form and women's aggression in a relational form (Björkqvist, 2018), it is difficult to predict how aggression will be manifested in online interactions. Wright (2020) reported that boys and girls with high masculine traits tend to express verbal aggression in online games, whereas boys and girls with high feminine traits express relational aggression in social media, suggesting that the differences in the dispositional types of aggression also apply to online spaces. Bettencourt and Miller's (1996) meta-analysis reported that gender differences in aggression decline under provocative situations; in online spaces with high provocations, women can also be as aggressive as men, making their circumstantial influences more prominent than dispositions.

However, the association between men's aggression and sexual behaviors is notable here. As men's aggression is biologically intertwined with reproductive behaviors (Cunningham et al., 2012), the increased aggression *per se* may indicate the increased risk of aggressive or hostile sexual behaviors. Such behaviors are manifested as the consumption of aggressive pornography as discussed above (Bridges et al., 2010; Fritz et al., 2020), and presumably, sexually offensive or hostile speech toward women on the Internet as well (Döring and Mohseni, 2019). However, the straight associations between the fundamental predispositions of males (e.g., hormone activities) and aggression/sexual behaviors might be weak or even trivial. Although Gallup et al. (2007) reported that men's handgrip strength relates to aggression and sexual behaviors, O'Connor et al.'s (2004) experimental study showed that testosterone injections among men do not directly increase aggressive or sexual behaviors, despite some increases in anger and hostility. However, it seems relatively more obvious that excessive masculinity as a personal attitude or a cultural norm can increase sexual aggression (Murnen et al., 2002). Locke and Mahalik (2005) demonstrated that male university students' belief in masculinity norms is associated with rape myth acceptance and sexual aggression. Such excessive or improper beliefs in masculinity are considered important components in the theories of aggressive sexual behaviors, including the attitudes of hostility toward women (Murnen et al., 2002; Malamuth and Hald, 2017). Nevertheless, the causation among aggressive predispositions, masculinity norms, and actual aggressive behaviors against women is still unclear, requiring further investigations in this field.

### Pseudo-rationalism

As discussed above, the uniqueness of Internet environments has made them full of people pursuing superiority over others. When such an attribute applies to the domain of judgment and decision-making, it may lead to the virtue of logically winning over others in online spaces. Moreover, as online spaces require rapid judgment rather than deliberation, such competition is likely to result in the “*I am right, you are wrong*” mindset, rather than true logical debates. These attitudes, now popular in most online communities, have the following beliefs in common: (i) reason or logic is superior to sensibility or empathy, (ii) their judgments are rational decisions based on logic, and (iii) the judgments of others that oppose their own are usually based on sentiments and are therefore irrational. The quote by Shapiro (2019), a famous right-wing commentator, is illustrative: his Twitter profile is famous with a pinned message of “*Facts don't care about your feelings*.” This is also common among young Korean anti-feminist men, which can be represented by Lee Jun-Seok's recent accusatory remark about subway protests by people with disabilities (as mentioned above) that they are “appealing to emotion, not reason” (Kang, 2022b). The alleged “rationalism” they propose is, in fact, diametrically opposed to true rationalism. Popper (1945) said that true rationalism understands the limitations of human knowledge and intelligence, admits that people can make mistakes in judgment and decision-making, recognizes how much people are indebted to others for knowledge, and therefore, does not hold impossible or unrealistic expectations from reason. In contrast, the “pseudo-rationalists” immodestly believe their intellectual superiority, pursue certain and immaculate truth, and eventually adopt rigid and authoritarian beliefs.

From a cognitive view, their attitudes can be explained by two concepts. The first is the Dunning-Kruger effect, i.e., the *inverse* correlation between one's competence and their confidence in their own competence (Dunning, 2011; Mahmood, 2016). This exhibits the paradoxical nature of metacognition that ignorant people are also more unaware of their own ignorance. Despite some criticisms regarding the reality of this phenomenon (e.g., Gignac and Zajenkowski, 2020), recent research shows that the Dunning-Kruger effect is also applicable to rigidity/dogmatism, prejudice, and extremism (Anson, 2018). West and Eaton (2019) argued that the Dunning-Kruger effect is also valid for the self-awareness of their own racism and sexism, demonstrating that those with stronger racist and sexist views also tend to disavow their prejudice more. Muller et al.'s (2021) neuroscientific study concluded that those who overestimate their own task performances tend to judge based on the process related to familiarity rather than the recollection-based process. This may be compelling evidence linking this effect to prejudice, considering the association between prejudice and the *status quo* bias (Jost, 2019). The second is the bias blind spot, the tendency to believe that cognitive bias pertains to others but not to themselves (Pronin et al., 2002). This phenomenon explains why people with bias also tend to derogate others more. Considering that this phenomenon refers to the denial of one's own bias and the exaggeration of others' bias, this may also be construed as a cognitive reinterpretation of projection, which was a famous psychodynamic mechanism but is now also empirically evident (Baumeister et al., 1998). Wang and Jeon (2020) demonstrated that bias blind spot also exists in various social stigmas and suggested that recognizing their own social bias can reduce people's prejudice.

Theoretically, however, the cognitive tendencies above are merely considered the measurable descendants of the motives related to prejudice rather than the origin of prejudice. According to Ross and Ward (1996), people tend to believe that they see the world objectively, which contributes to various social conflicts and misunderstandings. People think what they see is an objective fact, which must be seen by others as well (Ross et al., 1977), and when these beliefs are broken (i.e., when encountering people who think differently from themselves), they tend to believe that such people are senseless or biased. Therefore, the phenomenon of pseudo-rationality is based on people's fundamental self-centered bias, which is an elemental social-cognitive mechanism that regulates attitude formations and individual/social behaviors. In other words, this is an active motivation to confirm their beliefs, pursue certainty, and eventually perpetuate their ignorance (see Jost et al., 2003). Nevertheless, despite some theoretical background related to the metacognitive basis of pseudo-rationalism, empirical research regarding the effects of the “illusion of rationality” is lacking. Jung (2021) recently developed a scale to measure pseudo-rationalism and presented some correlations of this scale with dogmatism, perspective-taking, and intellectual humility.

## Group polarization/extremity

The development process of polarized identities as a consequence of group interactions can explain most extremist ideologies as well as anti-feminism, including those cultivated in online spaces (Vergani et al., 2020). This can be seen to be the final piece of the puzzle in manifesting extreme ideologies as a group identity, interacting with many individual or situational factors introduced above. Gaudette et al.'s (2020) qualitative study interviewed ten former online extremists and showed that the Internet is involved in the entire process of formation and development of extremism. Their narratives share some common stages: (i) being exposed to extremist ideologies through the Internet, (ii) starting interactions with extremists online, (iii) gradually beginning to sympathize with them, (iv) soothing loneliness and feeling a sense of belonging with them, (v) immersing themselves in online groups, and (vi) ultimately becoming self-sufficient for violent behaviors. Mostly, they were psychologically vulnerable to extremist ideologies, and the provocative claims online were enough to captivate their vulnerable hearts. Looking at these features, the theories of attachment insecurity or ostracism seem the most predominant for online extremist ideologies. However, it is notable that many of them were not new to extremist claims online, nor were they captivated by such claims in the first place. Their sympathy for extremist arguments was influenced not only by their own psychological vulnerability but also by the unique features of online spaces, as listed above, which led to extremist arguments being repeatedly reproduced and even seeming attractive. Although extremist claims might start as small voices with few sympathizers, continued exposure to these claims can gradually make people accept them, even including those who were initially opposed to such claims, due to the mere exposure effect (Zajonc, 1968; Bornstein and Craver-Lemley, 2017) or the sleeper effect (Lariscy and Tinkham, 1999; Kumkale and Albarracín, 2004). Finally, the agreement exponentially accelerates to become a huge social cascade (Sunstein, 2002; Wang et al., 2018).

When a particular group starts to conform to a particularly lean argument, people in the group start to form a homogenous group identity. Individually, they ignore the empirical evidence contrary to their thoughts (Brandt et al., 2015) and selectively accept weak supporting evidence or personal anecdotes, including disinformation (Nickerson, 1998; Ray and George, 2019). Collectively, the group influences its members to maintain a single thought, forming group norms and adjusting their attitudes according to the norms (Marques et al., 1998; Sunstein, 2002). Such a phenomenon becomes especially prominent when the members confront or perceive the opponent groups with contrasting ideologies to themselves, which bolsters their group identity (Hogg et al., 1990). Indeed, polarization is more likely to occur in the presence of opposing groups, such as anti-feminist backlash. Group interactions tend to be especially radical and hostile in online spaces. Yardi and Boyd (2010) argued that in social media, people reinforce the group identity when they see the arguments supportive of their groups, but when they encounter opposing arguments, they rather strengthen their exclusive attitudes to such arguments. The nature of the online space makes it easier for people to selectively accept specific information and act only in the direction of reinforcing their existing attitudes (Bessi et al., 2016). In addition to the users' opportunity to choose the information themselves, Internet service providers also play a role in reinforcing the users' existing positions by offering personalized content (Pariser, 2011; Bryant, 2020). Over time, they self-amplify their assertions within the exclusive room of homogeneous opinions or ideologies (“echo chambers”: O'Hara and Stevens, 2015), letting those with minority opinions exit the group (Sunstein, 2002). Finally, they evolve into an extremist group with a convergent polarized ideology (Atari et al., 2022).

It seems relatively obvious that Korean anti-feminism and male-victim ideology have passed through these processes of group polarization, developing their claims into more exclusive and homogeneous ones. Choo (2021) identified a strong association between anti-feminism and the usage of exclusive male communities among young Korean men. However, important to note is that such a process can apply to every intergroup dynamic and conflict, including online communities of young women. For example, Song and Kang (2018) concluded that exclusive attitudes were also observed in a radical online community of young women in Korea. Although some scholars argue that extreme ideologies or themes are more predominant in online anti-feminism (e.g., Rothermel, 2020), a study reported that polarized attitudes were observed among online feminist groups as well (Peña-Fernández et al., 2023), indicating that everybody is vulnerable to group polarization. It seems that the group interaction process only determines the severity of extremism, not its direction. Nevertheless, it can still explicate why young Korean men have been seen to have astonishingly extreme and homogeneous identities regarding gender issues. For young generations today, the Internet serves as a kind of huge peer group (Lehdonvirta and Räsänen, 2011). Young Korean men have been exposed to antagonistic claims about feminism on the Internet since they were very young (Jeong, 2013; Lee, 2013) and naturally learned extreme anti-feminist claims from their online peers, easily sympathizing with them (Kim and Lee, 2017). Therefore, in addition to their strong congruence and group identity, they tend to rely more on their online peers for judgment and decision-making, which is also an important component of group polarization (Boyd, 2023).

## Conclusion

Anti-feminism and male-victim ideology among South young Korean men have emerged relatively recently, despite their strong ramifications. Despite the lack of previous academic or psychological studies regarding this issue, psychological studies regarding modern racism and sexism outside Korea were discussed, which show very similar ideological features to the current anti-feminism in Korea. Through various types of data, it was evidenced that like other modern prejudices, Korean anti-feminism can also be considered general prejudice, rather than a unique phenomenon that applies only to specific concerns. Going further, multiple candidates for the antecedents of Korean anti-feminism that may trigger or accelerate hostility toward women or feminists were discussed, roughly based on the threat-defense theory, with evidence that supports or negates the plausibility of such candidates. This study is considered the deepest exploration of the psychological origins of Korean anti-feminism and male-victim ideology. This study adopted a multi-perspective approach regarding the complex social issue, utilizing various theories in psychological fields. Finally, I tried to reach a holistic conclusion, which is as cogent as possible given our current knowledge. I believe this interpretation and its all-inclusive approach can also provide insights into other prejudices or extremism discovered in various cultures and societies. This study shows that the theories regarding modern prejudice, mostly studied in the United States or Western societies, can also apply to Korean society, and such ideologies have some common psychological underpinnings irrespective of cultural backgrounds. Therefore, in the opposite direction, it would also be possible to apply the characteristics of online anti-feminism in Korea to sexist prejudice, online extremism, or general prejudice in other countries.

However, due to the scarcity of academic research and empirical evidence, this study could not reveal most of the mechanisms that form the specific ideological features, such as victim mentality. Although victim ideology has been observed in Western cultures as well, further studies are needed to identify its psychological antecedents. Furthermore, in this study, explanations of the Korean states mostly depended on unpublished reports: Although the poll results or descriptive statistics are not considered unreliable, some theories seem to need future academic validations. In addition, this study has developed its logic mainly through comparisons using generation and age, following the generational discourse adopted by Chun and Jeong (2019). Many parts of this study overgeneralized the uniqueness of “young men” themselves rather than anti-feminists only, mainly due to the paucity of evidence. Future studies must delve deeper into the individual characteristics of anti-feminism and male-victim ideology, as prejudice is not the exclusive property of a particular group or generation.

Moreover, the theories presented here are merely at the hypothetical stage, requiring verification through future studies. As the factors proposed in this study cover massive and extensive areas in social psychology, it seems that further studies should verify such potential factors individually rather than through all-inclusive research. The possible methodology includes correlational studies with self-report measures, experimental and neuroscientific studies, and analyses of social media to identify the features of interactions made in online spaces with quantitative modeling. Further studies should also consider the realm of Korean anti-feminism that the model proposed in this study does not explain. As this study has focused mostly on individual cognition and motivations, the sociocultural contexts that may influence modern sexism and extremism may have been ignored. Such contexts include social norms such as patriarchal values; although the effect of benevolent sexism is not observed among young Korean men in current studies, the potential influences of sexist social norms on the process of formulating their ideologies cannot be overlooked. Further studies could reveal the relationships between modern sexual prejudice in Korea or other countries and the cultural norms underlying it, such as patriarchal values or subtle gender stereotypes.

Above all, even if this model is later evidenced, it does not, by itself, indicate that Korean anti-feminism and male-victim ideology are completely a false consciousness. Their anti-feminist ideologies may have many different causes, some of which seem justifiable or admittable. However, at the very least, it seems obvious that certain psychological motives have facilitated their anti-feminism and male-victim ideology or inflated the severity of their extremity or its expressions. Moreover, despite some uniqueness, the primordial frameworks of these attitudes are identical to those of other forms of prejudice. As has been sparsely explained, this is a hallmark of modern extremism on a global scale, and contrary to what many people still believe, it has no cultural boundaries. The current society compels a deeper understanding of contemporary prejudice around the world. We should open our eyes to what is happening here and now, or more accurately, everywhere and now, and get closer to the implications of contemporary extremism and prejudice.

## Author contributions

The author confirms being the sole contributor of this work and has approved it for publication.
